# Wireless Inchworm-like Compact Soft Robot by Induction Heating of Magnetic Composite

**DOI:** 10.3390/mi14010162

**Published:** 2023-01-08

**Authors:** Woojun Jung, Seonghyeon Lee, Yongha Hwang

**Affiliations:** Department of Control and Instrumentation Engineering, Korea University, Sejong 30019, Republic of Korea

**Keywords:** wireless robot, soft robot, 3D printing, magnetic induction

## Abstract

Microrobots and nanorobots have been produced with various nature-inspired soft materials and operating mechanisms. However, freely operating a wirelessly miniaturized soft robot remains a challenge. In this study, a wireless crawling compact soft robot using induction heating was developed. The magnetic composite heater built into the robot was heated wirelessly via induction heating, causing a phase change in the working fluid surrounding the heater. The pressure generated from the evaporated fluid induces the bending of the robot, which is composed of elastomers. During one cycle of bending by heating and shrinking by cooling, the difference in the frictional force between the two legs of the robot causes it to move forward. This robot moved 7240 μm, representing 103% of its body length, over nine repetitions. Because the robot’s surface is made of biocompatible materials, it offers new possibilities for a soft exploratory microrobot that can be used inside a living body or in a narrow pipe.

## 1. Introduction

Microscale and nanoscale robots are being investigated for applications in the medical and biotechnology fields, such as drug delivery and noninvasive surgeries [1,2,3,4]. In particular, robots composed of soft materials have promising potential for tasks that require in vivo movement because they are flexible and stable owing to smooth interactions with the surrounding terrain [5]. These microscale soft robots are manufactured by casting soft polymers with biocompatibility and mechanical elasticity, such as polydimethylsiloxane (PDMS) and Ecoflex, into three-dimensional (3D) printed molds [6,7]. Even though PDMS, which is required as a material of actuator among these, has rather poor flexibility, it has been used for biomedical applications involving biological specifications, thereby proving its biocompatibility over the past decades [8]. The 3D printing mold casting technique enables rapid prototyping, facilitating the production of soft robots with various mechanisms and designs [9,10].

Several actuation mechanisms (such as pneumatic, thermal, piezoelectric, chemical, light-driven, and magnetic actuation) for small soft robots have been investigated. The soft pneumatic robot is deformed by applying air pressure through a tube connected to a space inside the soft body. Therefore, for pneumatic supply, an external air pump larger than the robot must be connected to the robot and tube [11,12,13]. Thermally operated soft robots mainly contain shape memory alloy (SMA) elements [14,15,16,17]. Generally, a Joule heating method is used to heat an SMA connected by a power supply and electric wires. Piezoelectric soft robots have built-in piezoelectric materials that exhibit mechanical deformation in response to electrical stimulation; therefore, power supply connections are required to apply high voltages of several hundreds of volts [18,19]. These methods, which require a physical connection using an external device, pose an obstacle to configuring a robot as a portable system and limit the distance it can move.

In contrast, a soft robot that operates via the chemical explosion of fuel mounted inside the robot can wirelessly operate because it does not require a connection to an external power supply [20,21]. However, because it operates based on fuel consumption inside the robot, it cannot operate continuously. A soft robot made of light-responsive materials can operate wirelessly; however, if an obstacle is encountered between the robot and the light source, the robot cannot function [22,23]. A soft robot whose movement is powered by magnetic forces can operate wirelessly even if an obstacle is encountered [24,25,26]. However, because magnetic materials are not soft, the robot can only express restricted flexible movements [24]. A soft robot combining magnetic particles and elastomers has been developed to solve this problem [25,27]; however, the deformation capacity is relatively low because the mechanical deformation of the elastomer by the magnetic field is expressed through the movement of the device. The magnetic induction method using Joule heating of the eddy current induced in magnetic materials through magnetic coils can be applied as an alternative to the wireless operation of robots [28]. This is because a significant deformation of a robot composed of elastomers can be induced by expanding the gas using heat generated in the magnetic particles by induction heating [29].

In this study, a wireless inchworm-like compact soft robot called WIBot (Wireless Inchworm-like robot) was developed using induction heating. Because the WIBot was formed by casting liquid PDMS into a 3D-printed mold, it was composed of a PDMS unibody without a bonding surface, fundamentally preventing internal fluid leakage [30]. A composite heater, formed by mixing Fe_3_O_4_ microparticles with uncured PDMS, was built inside the WIBot to achieve induction heating. A working fluid, methanol, was used to expand the WIBot. When an external magnetic field heated the composite heater, the internal pressure increased due to the evaporated methanol, expanding the WIBot membrane. Because the WIBot was designed such that the contact area of the two legs with the floor varied in three steps, it crawled in the intended direction with friction hysteresis during expansion and contraction.

## 2. Materials and Methods

### 2.1. Design

The aim of the robot design was to develop a soft robot that crawls wirelessly through repetitive movements and to examine its mechanism. Inspired by the movement of the inchworm, WIBot crawls by sequentially anchoring its front and rear legs during expansion and contraction. The crawling motion is stable and robust against the slope of the ground because the robot moves close to the ground rather than rolling or jumping.

**Figure 1:**
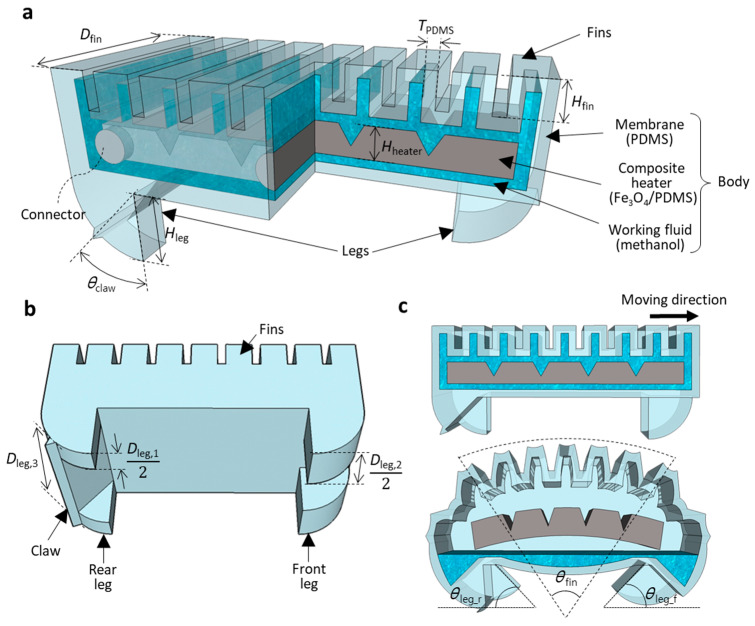
Overview of WIBot design. (**a**) Three-quarter section view of robot. Each leader line represents the robot’s components and materials. (**b**) Shape of robot’s legs for generating frictional force differences. (**c**) Description of relationship between legs and ground based on expansion and contraction of robot.

The WIBot consisted of body and leg parts (Figure 1). First, the body of the WIBot had an overall shape such that a membrane surrounded the internal space. Fins were placed on the upper surface of the body to enable the bending of the body as a result of expansion by pneumatic pressure. A heater was installed in the space inside the body, and the remaining space was filled with methanol. The Fe_3_O_4_/PDMS composite heater was heated via magnetic induction when an external magnetic field was applied to expand the 200 μm thick PDMS membrane (Table 1). As the methanol vaporized due to the heat supplied by the heater, the adjacent fins pushed each other away when they inflated by pressurizing the PDMS membrane, causing the body to bend significantly. Several grooves were formed in the composite heater to allow the robot to bend easily according to the bending direction. The composite heater was fixed by connecting it to the side of the PDMS membrane to prevent the expansion of the side surface of the PDMS membrane. Compared with ethanol, water, and paraffin wax, which have been used as phase-change materials because they have boiling points of 78 °C, 100 °C, and 370 °C, respectively, methanol has a relatively high specific energy of 22.034 MJ/kg and a low boiling point of 64.7 °C. Hence, methanol is an appropriate working fluid for compact soft robots [31] designed to operate in the range from room temperature to body temperature since there is no need to heat them to high temperatures. Since heat-induced changes in PDMS have been reported to be stable [32,33], the WIBot was designed using COMSOL numerical analysis with the second-order Ogden model for the PDMS, with the base and the agent mixed in ratio of 15:1 [34].

Second, the legs of the WIBot were designed in an arc shape along the trajectory of the bending of the body’s fins such that they slid smoothly. Three parts were designed with different tangent line lengths (i.e., depths) where the soles of the legs contacted the ground (Figure 1b,c). Thus, a difference existed in the applied frictional force between the leg and the ground based on the angle between the robot’s leg and the ground. This difference occurs because the maximum static frictional force increases with increasing contact area between the soft material surface and the ground [35].

**Figure 2:**
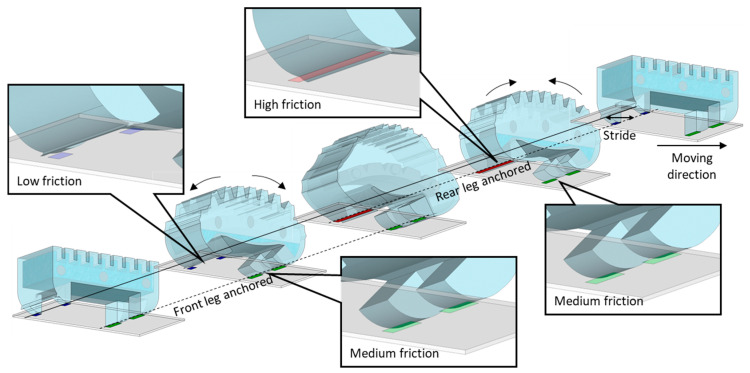
Moving mechanism of WIBot using friction hysteresis. When legs have tangential lengths of 1200, 2500, and 3500 μm, the substrate areas where the leg touches are marked blue, green, and red, respectively. The magnitude of the frictional force increases as the tangential length of the leg increases.

The front leg had a tangential length of 2300 μm; thus, a medium frictional force was generated among the three parts of the sole regardless of body bending. Conversely, the rear leg slid with a lower frictional force than the front leg because it had a tangential length of 1200 μm in the initial state. During heating, in which the WIBot moved from deflated to an inflated state, the frictional force of the front leg was higher than that of the rear leg. Thus, the front leg supported the body, whereas the rear leg slid and pulled forward. When the WIBot was inflated to the maximum, the part where the rear leg contacted the ground had a tangent length of 3500 μm, generating a greater frictional force than the front leg, which had a tangent length of 2300 μm. Consequently, during cooling, in which the WIBot returned from the inflated state to the deflated state, the rear leg supported the body because the frictional force of the rear leg exceeded that of the front leg; moreover, the front leg slid forward, causing the WIBot to crawl in the moving direction (Figure 2). The boundary line between the two areas, where the frictional force of the rear leg is different, was empirically set to 30° to the ground.

### 2.2. Fabrication

**Figure 3:**
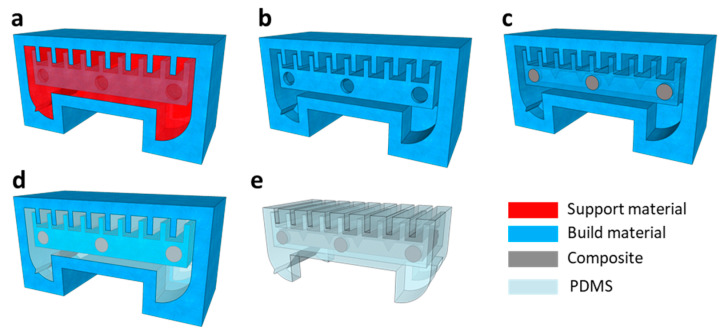
Fabrication process of WIBot using three-dimensional (3D)-printed soluble mold technique. (**a**) 3D-printed WIBot mold. (**b**) Mold with support material removed. (**c**) Injection of Fe_3_O_4_/PDMS composite only into inner mold and curing. (**d**) Casting of evenly mixed PDMS into mold. (**e**) Completed robot after mold dissolution (build material).

Figure 3 shows the fabrication process of the WIBot. First, the mold was printed with a computer-aided design using a 3D printer (3Z STUDIO, Solidscape, Merrimack, NH, USA) with a high resolution of 5 μm. A support material (Melt^TM^, Solidscape, USA) was automatically added to support the mold structure composed of the build material (Midas^TM^, Solidscape, USA) during lamination (Figure 3a). The support material was dissolved in a commercial dewax solvent (Bioact VSO, Petroferm, Gurnee, IL, USA) for 6 h at 55 °C (Figure 3b). The Fe_3_O_4_/PDMS composite had 65 wt% Fe_3_O_4_ magnetic microparticles (Daejung, Siheung-si, Republic of Korea) and 35 wt% uncured PDMS (Sylgard184, Dow Corning, Midland, MI, USA) uniformly mixed using a planetary mixer (AR-100, Thinky, Tokyo, Japan). Subsequently, the composite was loaded in a syringe and injected into the heater mold through a 550 μm hole in the center of the mold (Figure 3c). Six holes were formed in the heater mold to effectively dissolve the filled support material in the internal space. Four holes were sealed using PDMS patches before injecting the uncured Fe_3_O_4_/PDMS composite, and the other two holes were used as the injection ports for the composite. The composite was cured at 25 °C for 12 h. Next, the PDMS, with the base and the agent mixed in a ratio of 15:1 for low rigidity, was cast. The air bubbles between the uncured PDMS and mold were removed for 2 h in a vacuum chamber. The PDMS was cured for 6 h in an oven at 80 °C (Figure 3d). The mold filled with cured PDMS was immersed in acetone, which selectively melted only the build material (Figure 3e). After cleaning the cast device with deionized water, methanol (Daejung, Republic of Korea) was carefully injected as the working fluid into the membrane cavity using a syringe. Finally, after a small PDMS plug was placed in the hole formed by the syringe, the plug was fixed using an instant silicone adhesive. The fabricated robot size was 7 mm × 3.5 mm × 4 mm, including 1.2 mm long legs. The weight of the WIBot, including that of the working fluid, was 54 mg.

### 2.3. Measurement Setup

**Figure 4:**
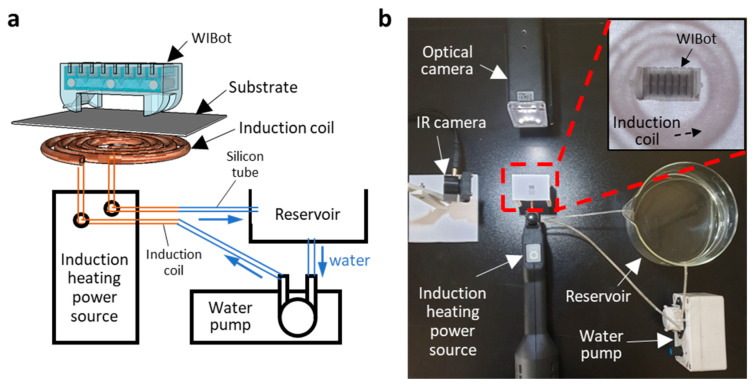
Experimental setup for WIBot. (**a**) WIBot placed on substrate. An induction coil made of a copper tube was connected to an induction-heating power source. Cold water is introduced into the coil to prevent temperature increase. (**b**) Photograph of experimental setup with main components labeled. A coil with an inner diameter of 8 mm and an outer diameter of 16 mm was made of a 1-mm-thick copper tube. The inset shows a close-up image of the WIBot on paper.

Figure 4 shows the measurement setup, consisting of a magnetic induction heater to operate the WIBot, an optical camera to capture the motion, and an infrared (IR) camera to simultaneously measure the temperature. The magnetic induction heater comprised a power source and an induction coil. An induction heating power source (Storm Heater, Bosjob, Taiwan, China) that could supply a frequency and voltage of 100 kHz and 16 V, respectively, generated a magnetic field by applying currents to the connected coil for induction heating of the composite heater of the WIBot. However, the current applied to the coil was accompanied by a temperature increase in the coil by Joule heating. A cooling system that passed cold water at 5 °C at a speed of 40 mL/min was required inside a copper tube coil to prevent the increase in coil temperature from affecting the WIBot temperature using methods other than induction heating. Finally, a paper substrate with a thickness of 100 μm was placed on the coil at intervals of 1 mm so that the WIBot could crawl.

## 3. Results and Discussion

**Figure 5:**
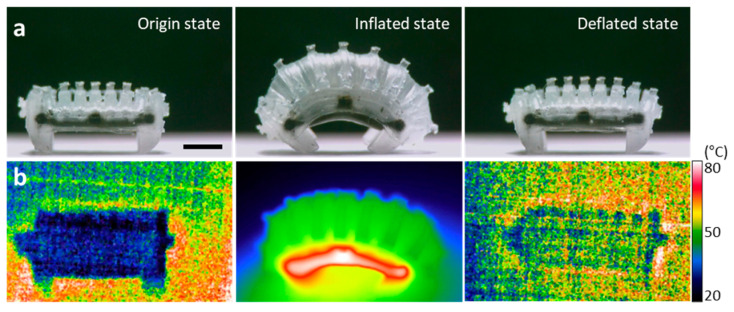
One-cycle operation process showing operation mechanism of WIBot. (**a**) Optical image showing movement distance of WIBot. The scale bar represents 2 mm. (**b**) Infrared image showing WIBot temperature.

The friction hysteresis of the legs generated during the expansion and contraction of the WIBot was manifested in its crawling movement. Figure 5 shows the experimental process, confirming that the WIBot crawled via friction hysteresis. A cycle of the WIBot operation consisted of 10 s of heating and 15 s of cooling, totaling 25 s (Figure 5a). The WIBot was maintained at room temperature (approximately 20 °C) in the inflated state, at which point the methanol inside the WIBot existed in a liquid state (Figure 5b). When an external alternating magnetic field was applied, the composite heater increased the temperature of the surrounding methanol, inducing a phase change. When the evaporated methanol pressurized the 200-μm-thick PDMS membrane, the expanded fins pushed the adjacent fins away, causing the WIBot to bend. The external magnetic field was maintained until the two legs of the WIBot reached a constant angle of 45° to the ground, and the heater was operated. At this time, it was presumed that the methanol, with a boiling point of 64.7 °C, vaporized inside the WIBot because the external surface temperature of the WIBot measured using the IR camera was 80 °C. During this process, the rear leg pulled forward because its frictional force was higher than that of the front leg.

When fully expanded, the part where the rear leg contacts the ground changed to the part with the maximum friction among the three leg parts. The heating inside the WIBot was discontinued by stopping the magnetic field application; thus, the WIBot was cooled via natural convection. During the cooling process, the rear leg was fixed while the front leg moved forward because the frictional force of the rear leg was greater than that of the front leg. As the methanol returned to a liquid state with decreasing temperature, the WIBot returned to the deflated state. Finally, the WIBot moved forward by a specific distance each time it underwent an expansion–contraction cycle. Here, the WIBot is not continuously cooled until it is restored to its complete initial state after the expansion. Instead, cooling is stopped after the contact surface of the rear leg changes from the high friction region to the low friction region, followed by the next repeated heating process. This is because the forward movement of the actuator is insignificant after the low friction area of the rear leg has touched to the floor. The external temperature does not critically change the operation time of the WIBot (Figure S1). After about 70 s, if the WIBot continues to cool until the body temperature decreases to room temperature, it returns to its initial shape. No serious plastic deformation was observed even after several operations, because the WIBot used a more flexible PDMS, in a ratio of 15:1, compared to the commonly used mix, which has base and agent mixed in a ratio of 10:1 [34].

**Figure 6:**
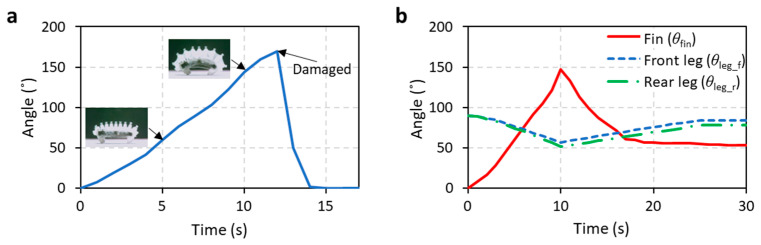
Measured fins and legs angles indicating appropriate heating and cooling time. (**a**) Arc angle formed by fins while WIBot is continuously heated. If the angle exceeds 160°, the robot incurs damage owing to excessive pressure. (**b**) Angles of fins and legs during heating for 10 s and subsequent cooling for 20 s. The leg angle represents the acute angle between the ground and the leg.

As the WIBot consisted of a PDMS membrane with a thickness of 200 μm, excessive expansion could potentially damage the PDMS membrane, leading to methanol leakage. The tensile strength of PDMS is 6.7 MPa [36]. However, the likelihood of tearing of the WIbot’s membrane due to excessive expansion varies not only with the material properties of PDMS but also according to various structural parameters such as the angle, shape, and thickness of fins and body. To identify the moment of excessive expansion, the WIBot was continuously heated to check when damage occurred (Figure 6a). The angle of the arc formed by the fins was measured until the WIBot was damaged by excessive expansion of the fins. On average, the WIBot broke at fin angles greater than 170°. Subsequently, the WIBot was heated for 10 s, the heating time at which the fin angle became 150°, taking into account the difference between samples, such that the WIBot could operate stably.

The following angles were evaluated to verify that the leg of the inflated WIBot was restored to the rest state (Figure 6b): the arc angle (θ*_fin_*) formed by the fins, the acute angle between the front leg and ground (θ*_leg_f_*), and the acute angle between the rear leg and ground (θ*_leg_r_*). The cooling mechanism and time are critical parameters for determining the operating speed. The WIBot was cooled via natural convection in ambient air. The θ*_leg_f_* and θ*_leg_r_* values of the WIBot were maintained at 90° in the original state. Subsequently, they decreased to 57° and 52°, respectively, during heating. The leg angles were then restored to the deflated state of 84° and 78° within 15 s, respectively, after cooling commenced. Thus, a cycle of the WIBot operation consisted of heating for 10 s and cooling for 15 s.

The change in the leg angle can help clarify the movement mechanism of the WIBot. As described in Section 2.1, friction hysteresis is passively activated because the rear leg of the WIBot is designed to have different contact lengths with the ground during expansion and contraction. The length of the tangent line where the rear leg touched the floor was divided into 1200 and 3500 μm, based on a θ*_leg_r_* value of 30°. Furthermore, a 200 μm high protruding sharp claw was designed at the boundary between the two regions. The claw was pressed onto the ground based on the direction in which θ*_leg_r_* varied (i.e., the direction in which the leg moved), delaying the transition between both parts. Thus, when the front leg was fixed while the rear leg moved forward during the expansion of the body, even at θ*_leg_r_* = 60°, the claw was pressed onto the ground, delaying the change in tangential length. However, when the body continued to expand, and θ*_leg_r_* became smaller than 55°, the claws came off the ground. At this moment, the tangent length at which the rear leg touched the floor changed from 1200 to 3500 μm. Therefore, when the rear leg was fixed while the front leg moved forward during body contraction, the claws pressed on the floor in the opposite direction of heating. When θ*_leg_r_* reached 52°, the rear leg maintained a tangential length of 3500 μm and was then converted to 1200 μm. Therefore, friction hysteresis of the WIBot occurs because the area in contact with the ground varies with the direction in which the rear leg moves (expansion or contraction of the body), causing the robot to move forward.

The temperature change of the WIBot gradually decreased as the distance from the coil decreased (Figure S2). When the distance between the coil and the WIBot became more than 6 mm, the WIBot was no longer operated by induction heating. However, the effective distance for the magnetic induction heating phenomenon is profoundly changed by several factors such as the voltage and frequency of the power source, the number of windings of coils, thickness of coils, arrangement of multi-coils, and material properties of the workpiece to be heated. In particular, the Helmholtz coil, which forms a nearly uniform magnetic field because it consists of two coils on the same axis, can improve the effective distance of the robot by induction heating.

**Figure 7:**
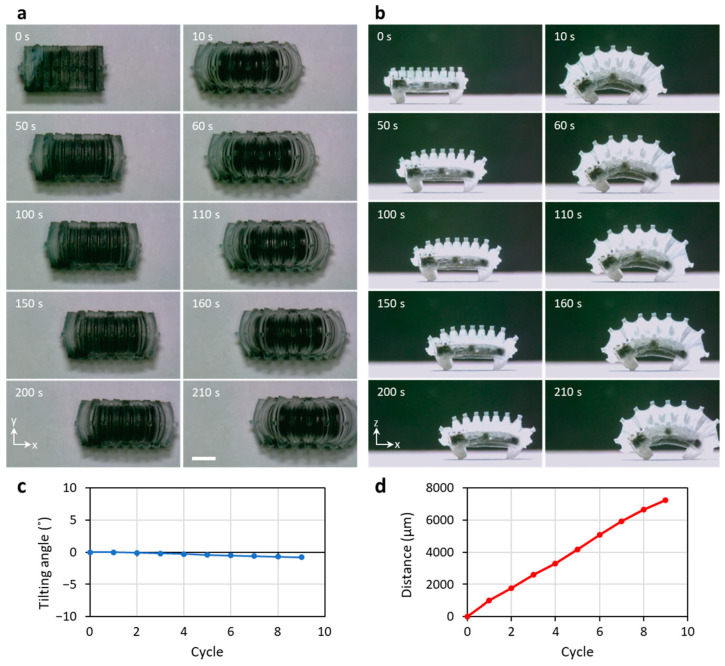
Demonstration of crawling based on repetitive motion of the WIBot. (**a**) Top view to check the WIBot is crawling in a straight line. (**b**) Side view to measure displacement of the WIBot. The scale bar represents 2 mm. (**c**) The tilting angle and (**d**) the moving distance according to repeated operation.

Figure 7 shows still cuts of the video of the robot crawling by continuous movement on a flat paper surface (roughness R_a_ = 10 μm) placed horizontally. Figure 7a shows a top view of the repetitive movement of the WIBot, which confirms that it moves in a straight line. The moving distance of the center point was evaluated using a Python-based tool on the experimentally recorded image to quantitatively measure the movement of the WIBot. After nine repetitions, the robot rotated 0.8° clockwise along the *x*-axis, which corresponded to the front direction of the WIBot, while moving at 7240 μm (Figure 7c,d). Therefore, the WIBot, whose side expansion was limited by the connection of the composite heater to the PDMS membrane, moved in its intended direction without tilting.

Figure 7b and Video S1 were filmed from the side view, showing the crawling movement through the repetitive operation of the WIBot. The WIBot moved to a length corresponding to 103% of its body length in nine repetitions. Additionally, the maximum fin angle was 146° on average, with a standard deviation of 2.1°, demonstrating consistent repeatability. The average stride length of the WIBot while crawling was 830 μm, with an average speed of 32.2 μm/s. The WIBot worked at an average of 3.84 μJ, with the energy received through wireless power transmission. Additionally, on glass with a surface roughness of 0.01 μm, the friction between the PDMS leg of the WIBot and the glass floor was too small, causing both legs to slip. Thus, the WIBot was observed to move 1270 um over four cycles by crawling, as the frictional forces of the front and rear legs did not differ during repeated expansion and contraction (Video S2). On a sandpaper with a surface roughness of 40 μm, the WIBot only crawled 2520 μm for four cycles due to excessive friction between the legs and the floor (Video S3).

In future studies, we intend to investigate the motion of a robot with a surface coating that adjusts the coefficient of friction on substrates with varying roughness, underwater, or in a windy outdoor environment. In addition, wireless robots that reduce the risk of heat can be developed through appropriate working fluids such as PF-5052 and Novec-7000 Engineered fluid from 3M^®®^ and accurate temperature control. Furthermore, instead of relying on natural convection, an active cooling system may increase the movement speed of the WIBot by reducing the recovery time after bending. We also expect that the arrangement of multiple coils and the delicate adjustment of each coil will allow the WIBot to undertake more complex movements such as direction and speed control.

## 4. Conclusions

In this study, we demonstrated the forward motion of a WIBot, a subgram-scale wireless inchworm-like compact soft robot equipped with a magnetic composite heater and a working fluid. The soft robot, which was composed of a unibody created using 3D printing technology, exhibited effective wireless movement via frictional hysteresis through geometric design. When the composite heater was heated by induction heating, the PDMS membrane expanded owing to atmospheric pressure as methanol, a working fluid with a low boiling point, evaporated. After the WIBot expanded fully, it was cooled via natural convection until its temperature reached room temperature. Finally, when methanol returned to the liquid state, the WIBot switched to the deflated state. The WIBot crawled by advancing 7240 μm through nine cyclic expansions and contractions. The developed WIBot used PDMS in its composition, a biocompatible material, which may be utilized as a foundation for a new generation of medical robot that can move inside a living body or a wireless microrobot for exploring narrow spaces, such as pipes.

## Figures and Tables

**Table 1:** Key dimensions of WIBot design. (Symbols are illustrated in Figure 1).

Symbol	Description	Value
*T*_PDMS_	PDMS thickness	0.2 mm
*H*_fin_	Fin height	0.6 mm
*D*_fin_	Fin depth	3.5 mm
*N*	Number of fins	9
*H*_heater_	Heater height	0.6 mm
*θ*_claw_	Claw angle	30°
*H*_leg_	Leg height	1.2 mm
*D*_leg,1_	Leg depth 1	1.2 mm
*D*_leg,2_	Leg depth 2	2.3 mm
*D*_leg,3_	Leg depth 3	3.5 mm

## Data Availability

The data presented in this study are available on request from the corresponding author.

## Supplementary Materials

The following supporting information can be downloaded at: https://www.mdpi.com/article/10.3390/mi14010162/s1, Figure S1: numerical analysis of the expansion of the WIBot; Figure S2: temperature change of the WIBot according to the air-gap distance between the WIBot and the induction coil; Video S1: repeated operation of WIBot on paper; Video S2: repeated operation of WIBot on glass; Video S3: repeated operation of WIBot on sandpaper.
