# Supplementary material for: Wireless Inchworm-like Compact Soft Robot by Induction Heating of Magnetic Composite
Source: Micromachines (Basel). 2023 Jan 8;14(1):162. doi: 10.3390/mi14010162 (PMC9865691; doi:10.3390/mi14010162)
Supplement: Supplementary file 1 [file micromachines-14-00162-s001.zip › Supplementary_materials.pdf]

Wireless Inchworm-like Compact Soft Robot by Induction Heating of Magnetic Composite

Woojun Jung, Seonghyeon Lee and Yongha Hwang*

Department of Control and Instrumentation Engineering, Korea University, Sejong 30019, Republic of Korea
* Correspondence: hwangyongha@korea.ac.kr

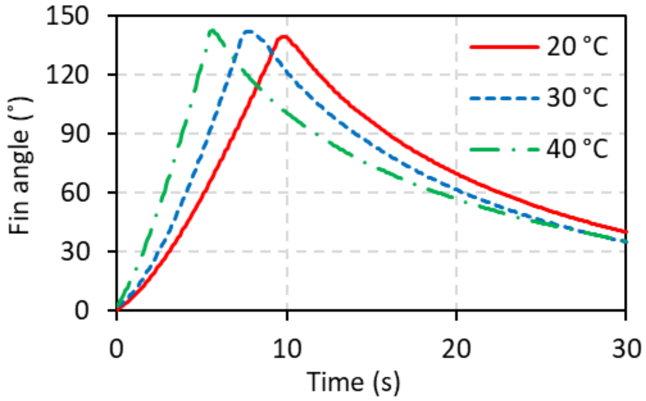

Figure S1. The numerical analysis of the expansion of the WIBot. When the ambient temperature outside the robot is increased from 20 °C to 30 °C and 40 °C, the time required for the temperature of the robot to be heated to 80° decreases from 10 s to 8 s and 6 s, respectively.

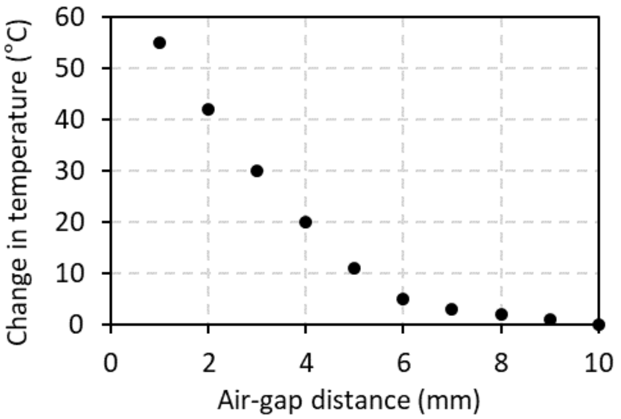

Figure S2. Temperature change of the WIBot according to the air-gap distance between the WIBot and the induction coil. When the distance between the coil and the WIBot become more than 6 mm, the WIBot is no longer operated by induction heating.
